# Safety of emergent carotid stenting after thrombolysis: a multicenter retrospective matched analysis

**DOI:** 10.1007/s00234-025-03571-8

**Published:** 2025-03-21

**Authors:** Francesca Colò, Andrea M. Alexandre, Valerio Brunetti, Francesco Arba, Luca Scarcia, Alessandro Pedicelli, Mariangela Piano, Maria Ruggiero, Joseph D. Gabrieli, Valerio Da Ros, Daniele Romano, Riccardo Russo, Anna Cavallini, Guido Bigliardi, Antonio A. Caragliano, Maria P. Ganimede, Giancarlo Salsano, Pietro Panni, Emilio Lozupone, Sabrina Anticoli, Monica Ferrante, Andrea Zini, Danilo Toni, Thanh N. Nguyen, Frédéric Clarençon, Aldobrando Broccolini, and the Collaborators of the emergent CArotid Artery Stenting (eCAS) study group

**Affiliations:** 1 School of Medicine, Catholic University of the Sacred Heart, Rome, Italy; 2 U.O.S.A. Neuroradiologia Interventistica, Fondazione Policlinico Universitario A. Gemelli IRCCS, Rome, Italy; 3 Neurology Unit, Fondazione Policlinico Universitario A. Gemelli IRCCS, Rome, Italy; 4 Stroke Unit, A.O.U. Careggi, Florence, Italy; 5 Neuroradiology Unit, Henri Mondor Hospital, Creteil, France; 6 Neuroradiology Unit, ASST Grande Ospedale Metropolitano Niguarda, Milan, Italy; 7 Interventional Neuroradiology Unit, AUSL Romagna, M. Bufalini Hospital, Cesena, Italy; 8 Neuroradiology Unit, Policlinico Universitario di Padova, Padua, Italy; 9 Department of Biomedicine and Prevention, University Hospital of Rome “Tor Vergata”, Rome, Italy; 10 Neuroradiology Unit, A.O.U. S Giovanni di Dio e Ruggi di Aragona, Salerno, Italy; 11 Neuroradiology Unit, A.O. Città della Salute e della Scienza, Turin, Italy; 12 Department of Emergency Neurology and Stroke Unit, IRCCS C. Mondino, Pavia, Italy; 13 Stroke Unit, Ospedale Civile di Baggiovara, Modena, Italy; 14 Neuroradiology Unit, A.O.U. Policlinico G. Martino, Messina, Italy; 15 Neuroradiology Unit, “SS Annunziata” Hospital, Taranto, Italy; 16 Neuroradiology Unit, IRCCS Ospedale Policlinico S. Martino, Genua, Italy; 17 Interventional Neuroradiology Unit, IRCCS San Raffaele University Hospital, Milan, Italy; 18 Neuroradiology Unit, Vito Fazzi Hospital, Lecce, Italy; 19 Stroke Unit, San Camillo Forlanini Hospital, Rome, Italy; 20 Department of Neurology and Stroke Center, IRCCS Istituto delle Scienze Neurologiche di Bologna, Maggiore Hospital, Bologna, Italy; 21 Emergency Department Stroke Unit, University Hospital Policlinico Umberto I, Rome, Italy; 22 Neurology, Radiology, Boston Medical Center, Boston, USA; 23 Department of Neuroadiology, Pitié-Salpetrière Hospital, Paris, France

**Keywords:** Tandem lesions, Emergent carotid artery stenting, Mechanical thrombectomy, Intravenous thrombolysis, Parenchymal hemorrhage

## Abstract

**Purpose:**

Mechanical thrombectomy (MT) with emergent carotid artery stenting (eCAS) has been suggested to provide greater benefits for patients with tandem lesions (TL), but there is uncertainty about the most appropriate peri-procedural antiplatelet therapy for patients at higher risk of brain hemorrhage. This study aimed to assess the safety of intravenous thrombolysis (IVT) in patients with acute TL undergoing MT with eCAS.

**Methods:**

The databases of 17 stroke centers were retrospectively screened for consecutive patients with acute TL who underwent MT and eCAS. Propensity score matching (PSM) was used to evaluate the safety of IVT, balancing for peri-procedural antiplatelet therapies. Primary outcome measures were the occurrence of parenchymal hemorrhage (PH) type 2 and mortality within 90 days from the index event. Secondary outcome measures included occurrence of PH type 1, extracranial bleeding events, early stent thrombosis, efficient recanalization after MT and the 90-day functional outcome.

**Results:**

Among 560 enrolled patients, 47.3% received IVT prior to the endovascular procedure. After PSM, there was no significant difference between patients treated with and without IVT under different antiplatelet regimens concerning the rates of PH type 2 (5.2% versus 6.9%, *p* = 0.7, respectively) and of mortality of any cause (7.5% vs. 8.2%, *p* = 0.8). In addition, IVT did not impact recanalization rates or clinical outcome.

**Conclusions:**

The safety of MT with eCAS in acute TL is not affected by prior IVT. Furthermore, IVT does not ameliorate recanalization rates and clinical outcome. These findings are exploratory and require validation through future randomized controlled studies.

**Supplementary Information:**

The online version contains supplementary material available at 10.1007/s00234-025-03571-8.

## Introduction

Tandem lesions (TL), defined as high-grade stenosis or occlusion of the cervical internal carotid artery (ICA) and concurrent ipsilateral intracranial large vessel occlusion (LVO) in the anterior circulation, constitute 10–15% of all LVO acute ischemic strokes [1]. Mechanical thrombectomy (MT) with emergent carotid stenting (eCAS) has been suggested to provide benefit in TL treatment, although there is incomplete agreement on the peri-procedural antithrombotic treatment to balance between efficacy and safety [2–5]. First-line agents include i.v. aspirin, alone or in combination with clopidogrel, and glycoprotein IIb/IIIa inhibitors (GPI), but other antiplatelet drugs are also used [3, 6, 7]. One of the main concerns regarding eCAS is the risk of intracerebral hemorrhage associated with the peri-procedural antiplatelet therapy needed to promote stent patency. In such a scenario, previous use of intravenous thrombolysis (IVT) can make the neurointerventionalist less inclined to perform stenting [8]. Indeed, there is evidence that patients who received IVT are at increased risk of intracranial hemorrhage following early administration of antithrombotic agents, as might be the case during eCAS [9]. On the other hand, although current guidelines recommend to start antiplatelet therapy 24 h after IVT, it is possible to overcome this limit in cases when withholding such treatment can lead to a substantial risk [10]. Since in real-world practice peri-procedural protocols are heterogeneous in terms of type and combination of antithrombotic agents, in the absence of clear indications there may be a tendency to avoid a more aggressive antiplatelet treatment, or even prefer angioplasty without stenting, in patients deemed at higher risk for bleeding [8].

This study aimed to assess whether IVT affects the safety and efficacy of MT eCAS, after adjustments for the peri-procedural antiplatelet regimens and other baseline clinical factors. Efficacy outcomes were assessed as a secondary aim.

The analysis was conducted in adherence with the STrengthening the Reporting of OBservational studies in Epidemiology (STROBE) statement [11].

## Methods

### Patients and treatments

In this retrospective observational study, the prospective databases of 17 comprehensive stroke centers were screened for consecutive patients with TL who received MT with eCAS between January 2016 and June 2023. Demographics and baseline clinical and imaging data were collected. Patients were diagnosed with a non-contrast computed tomography (CT) scan followed by CT angiography to locate the site of occlusion. Tandem lesion was defined as a combination of a severe stenosis or occlusion of the extracranial ICA and occlusion of the M1 segment or proximal M2 segment of the MCA, according to the criteria adopted in the Thrombectomy in Tandem Lesion study [12]. Intravenous thrombolysis with tissue plasminogen activator was administered when the patient was eligible [10]. The different peri-procedural antiplatelet regimens for eCAS were recorded.

Endovascular procedures were performed via a transfemoral or trans-radial approach. In brief, an 8 F guiding catheter or an 8–9 F balloon-guiding catheter was introduced into the common carotid artery and an angiographic run was performed to evaluate the occlusion (atherothrombotic versus dissection). The cervical ICA treatment approach (antegrade or retrograde) was at the discretion of the neurointerventionalist and included stenting with or without angioplasty. Mechanical thrombectomy was performed with a stent-retriever, contact aspiration or using a combined technique. A score of 2b-3 in the modified Treatment in Cerebral Infarction (mTICI) scale was the measure of successful recanalization [13]. All procedures were conducted under general anesthesia or local anesthesia/conscious sedation, according to the local protocol or at the discretion of the treating physician. Stent patency was assessed within 24 h using ultrasound or CT angiography.

In each participating center, two neuroradiologists with more than five years of experience, who were blinded to procedural and outcome records, reviewed all radiological and angiographic data of their patients. In cases of doubt or disagreement, re-evaluation and adjudication were performed through consultation.

### Measures of outcome

Intracranial hemorrhagic events were assessed on follow-up imaging within 72 h post-procedure. Clinical outcome was assessed using the modified Rankin Scale (mRS) score, obtained 90 days after the index event by a trained physician during a scheduled visit or via telephone interview.

Primary safety outcome measures were: (1) the occurrence of brain parenchymal hematoma (PH) type 2, defined as a PH occupying > 30% of the infarcted tissue with relevant mass effect, according to the Heidelberg classification of bleeding events after reperfusion therapies [14], and (2) mortality from any cause within 90 days from the index event. Secondary safety outcome measures included the occurrence of (1) PH type 1 (PH occupying < 30% of the infarcted tissue with no or minimal mass effect), (2) major extracranial bleeding (any bleeding event requiring immediate medical intervention), and (3) early stent thrombosis, defined as thrombosis occurring within 24 h after the endovascular procedure.

Efficacy outcome measures included: (1) successful recanalization at the end of endovascular treatment, and (2) 90-day functional independence, defined as an mRS score of 0–2.

### Statistical analysis

Descriptive statistics were used to define baseline characteristics. The study population was divided into two groups based on whether they had received IVT before the endovascular treatment (IVT and no-IVT groups). Differences between categorical variables were compared using Fisher’s exact test, whereas continuous variables were compared using the Welch two-sample t-test or Mann-Whitney U test according to their distribution. The Shapiro-Wilk test was used to test the normality of continuous variables. Missing values were not imputed. Significance threshold was set at p-value < 0.05.

Because our patients were not randomized, we used propensity score matching (PSM) to estimate the difference in outcome measures between IVT and no-IVT patients. This was done to minimize the effect of baseline clinical and procedural features. Therefore, covariates for the matching algorithm were chosen in accordance with their potential role on safety and clinical outcomes measures and included age, the baseline National Institutes of Health Stroke Scale (NIHSS) score, the baseline Alberta Stroke Program Early CT score (ASPECTS), prior antiplatelet or anticoagulant therapies, the type of peri-procedural antiplatelet therapy for eCAS and the onset-to-groin (OTG) time. The “greedy nearest-neighbor” method was used to create 1:1 pairs of patients that had very similar propensity scores, setting a caliper width of 0.02 of the propensity score scale. propensity score matching balance was assessed by checking the standardized mean difference (SMD) between covariates with a value < 0.1 indicating negligible imbalance [15].

Subgroup matched analyses were conducted after separation of patients according to the type of peri-procedural antiplatelet therapy, if this was possible for the size of each treatment group and using the same covariates. A descriptive comparison of outcomes was instead performed for those groups of treatment having a number of patients not sufficient to perform PSM.

All analyses were performed using the R software v.4.3.2 (https://www.r-project.org).

## Results

A total of 560 consecutive patients (174 female, 31.1%) with TL who underwent MT and eCAS were enrolled. Two hundred sixty-five patients (47.3%) received IVT prior to the endovascular procedure. Atheromatous disease of the cervical ICA was present in 82.1% of patients. Successful recanalization was achieved in 481 patients (87.1%). Occurrence of PH type 2 was present in 43 patients (7.7%), whereas PH type 1 occurred in 138 (24.6%).

The two most frequent peri-procedural antiplatelet regimens were (1) i.v. aspirin 250–500 mg only (119 patients, 21.2%) or (2) GPI loading dose (i.v. tirofiban 12 µg/kg in 30 min or i.v. abciximab 0.25 mg/kg) followed by a GPI maintenance dose (i.v. tirofiban 0.1 µg/kg/min or i.v. abciximab 0.125 µg/kg/min) over 12 h after the endovascular treatment (204 patients, 36.4%). Other antiplatelet regimens were: (1) i.v. aspirin 250–500 mg followed by a GPI maintenance dose over 12 h after the endovascular procedure (51 patients, 9.1%), (2) i.v. cangrelor (30 µg/kg bolus + 4 µg/kg/min i.v. for 2 h; 62 patients, 11.1%), or (3) i.v. aspirin 250–500 mg plus clopidogrel 75–500 mg (76 patients, 13.6%).

A minority of patients received either (1) i.v. GPI loading dose only (20 patients, 3.6%), (2) i.v. cangrelor followed by GPI maintenance (9 patients, 1.6%), or (3) dual antiplatelet therapy with i.v. aspirin and clopidogrel followed by GPI maintenance (7 patients, 1.2%). Twelve patients (2.1%) did not receive any peri-procedural antiplatelet therapy.

Univariate analysis of clinical and neuroradiological features, peri-procedural antiplatelet therapies, and outcome data of the two raw groups is reported in Supplementary Tables 1 and 2.

Propensity score matching of the entire population yielded 174 couples of patients balanced for age, NIHSS score and ASPECTS at baseline, prior antiplatelet or anticoagulant therapy, type of peri-procedural antiplatelet therapy and OTG time. Standardized mean difference values between covariates and the distribution of the different peri-procedural antiplatelet therapies in the post-match populations are reported in Supplementary Tables 3 and 4. There was no significant difference between patients treated with and without IVT under different antiplatelet regimens concerning the rates of PH type 2 (5.2% versus 6.9%, *p* = 0.7) and mortality of any cause (7.5% vs. 8.2%, *p* = 0.8). Similarly, we did not find any difference concerning rates of PH type 1 (27.0% in the IVT groups vs. 25.3% in the no-IVT group, *p* = 0.8), of major extracranial bleeding events (0.5% vs. 0.0%, *p* = 0.5) and of early stent thrombosis (5.7% vs. 6.3%, *p* = 1). There was also no significant effect of prior IVT on rates of efficient recanalization and 90-day functional independence (Table 1).

**Table 1:** Outcome measures after PSM of IVT and no-IVT patients under different peri-procedural antiplatelet therapies

	IVT (*N*=174)	no-IVT (*N*=174)	*p* *
PH type 2, n/N (%)	9/174 (5.2%)	12/174 (6.9%)	0.7
90-day mortality, number/total (%)	12/159 (7.5%)	13/158 (8.2%)	0.8
PH type 1, n/N (%)	47/174 (27.0%)	44/174 (25.3%)	0.8
major extracranial bleeding events, n/N (%)	1/174 (0.5%)	0/174 (0.0%)	0.5
early stent thrombosis, n/N (%)	10/174 (5.7%)	11/174 (6.3%)	1.0
mTICI score 2b-3, n/N (%)	145/174 (83.3%)	154/174 (88.5%)	0.2
90-day mRS score 0-2, n/N (%)	100/159 (62.9%)	91/158 (57.6%)	0.4

PSM = propensity score matching; IVT = intravenous thrombolysis; PH = parenchymal hemorrhage; mTICI = modified Treatment In Cerebral Infarction; mRS = modified Rankin Scale; * statistical significance was considered at *p* < 0.05

Subgroup matched-pair analysis could be done only for patients who received peri-procedural aspirin and those receiving GPI loading dose plus GPI maintenance. Standardized mean difference between covariates before and after PSM are reported in Supplementary Tables 5 and 6. Similarly to what was observed in the comprehensive matched analysis, for both these subgroups there was no significant difference in the occurrence of PH type 2 and rates of 90-day mortality between IVT and no-IVT patients. There was also no significant difference regarding the other safety and efficacy outcome measures (Table 2).

**Table 2:** Outcome measures after PSM of IVT and no-IVT patients under peri-procedural I.v. Aspirin or I.v. GPI loading dose + maintenance

	i.v. Aspirin			i.v. GPI loading dose + maintenance		
	**IVT (** ***N*** **= 26)**	**no-IVT (** ***N*** **= 26)**	*p* *	**IVT (** ***N*** **= 54)**	**no-IVT (** ***N*** **= 54)**	*p* *
PH type 2, n/N (%)	1/26 (3.8%)	3/26 (11.5%)	0.6	8/54 (14.8%)	3/54 (5.5%)	0.2
90-day mortality, n/N (%)	4/24 (16.7%)	1/25 (4.0%)	0.2	4/53 (7.5%)	5/53 (9.4%)	1.0
PH type 1, n/N (%)	9/26 (34.6%)	6/26 (23.1%)	0.5	8/54 (14.8%)	12/54 (22.2%)	0.5
major extracranial bleeding events, n/N (%) number/total (%)	0/26 (0.0%)	0/26 (0.0%)		0/54 (0.0%)	0/54 (0.0%)	
early stent thrombosis, n/N (%)	1/26 (3.8%)	0/26 (0.0%)	1.0	6/54 (11.1%)	6/54 (11.1%)	1.0
mTICI score 2b-3, n/N (%)	24/26 (92.3%)	23/26 (88.5%)	1.0	41/54 (75.9%)	45/54 (83.3%)	0.5
90-day mRS score 0-2, n/N (%)	13/24 (54.2%)	13/25 (52.0%)	1.0	30/53 (56.6%)	27/53 (51.0%)	0.7

PSM = propensity score matching; IVT = intravenous thrombolysis; GPI = glycoproteinIIb/IIIa inhibitor; PH = parenchymal hemorrhage; mTICI = modified Treatment In Cerebral Infarction; mRS = modified Rankin Scale; * statistical significance was considered at *p* < 0.05

A descriptive comparison of outcomes for patients under the less frequent peri-procedural antiplatelet regimens is reported in Supplementary Table 7. With the limitation connected with small and unadjusted samples, we did not find any difference in safety outcome measures between IVT and no-IVT patients.

## Discussion

Our multicenter retrospective matched analysis has shown that prior administration of IVT is not associated with increased rates of PH type 2 or 90-day mortality in patients undergoing MT with eCAS for acute TL, despite different peri-procedural antiplatelet regimens. Therefore, IVT should not be a reason to withhold acute stenting due to concerns over a cumulative risk of intracranial hemorrhage from early administration of antiplatelet agents required for stent patency. This evidence may be also valuable during management of stroke patients with other concomitant acute medical conditions, that may require a timely initiation of antiplatelet therapy despite prior thrombolysis.

Although MT and eCAS have been suggested to have a greater benefit compared to procedures without stenting, the risk of intracranial bleeding remains an important concern, mainly when considered after IVT [2, 5, 8]. Such concerns arise from the lack of data from dedicated trials and leads to a wide variability of peri-procedural antithrombotic protocols across different centers. Although other studies have suggested that prior IVT does not increase the risk of hemorrhage, they primarily grouped patients based on the intensity of the antithrombotic regimen used for eCAS rather than adjusting for specific peri-procedural therapies [3, 4, 16]. Our study aimed to address this limitation by tailoring the analysis to account for the individual antiplatelet regimens. However, while this approach was adequate for evaluating the overall effect of IVT, the relatively low number of patients receiving certain peri-procedural antiplatelet regimens limited our ability to assess meaningful differences in the safety effects of different agents in relation to IVT.

Our analysis confirmed the wide variability of peri-procedural antithrombotic therapies in real-world practice (with preference towards protocols using aspirin and/or GPIs). The most efficient antiplatelet regimen with a minimal risk of brain bleeding remains unclear, as current evidence is uneven, particularly regarding the use of GPIs. While beneficial for maintaining stent patency, GPI use of in different settings of acute stroke management is controversial due to a possible increased risk of intracranial hemorrhage or fatal brain bleeding events [17–19]. On the other hand, it has been recently shown that peri-procedural use of tirofiban is associated with a lower risk of in-stent thrombosis and symptomatic intracranial hemorrhage compared to aspirin [20]. Pending the results of randomized trial, it is conceivable that caution should be used when considering GPI administration. Indeed, because GPIs provide robust inhibition of fibrinogen binding to platelets, this mechanism may theoretically be relevant in patients previously treated with IVT, as such treatment can result in coagulopathy due to fibrinogen depletion [21, 22]. However, our subgroups matched analysis in patients receiving peri-procedural GPI did not show any significant effect of IVT on safety outcome measures. In this context, it is unclear whether the unusual association of other antiplatelet agents with GPI, as observed in our real-world analysis, can be justified and beneficial, without increasing the risk of adverse events. Concerning other peri-procedural regimens, cangrelor appears to carry a good efficient and safety profile, though comparative analyses focusing on prior IVT administration are lacking [23, 24]. Unfortunately, our analysis failed to provide meaningful information on specific regimens that were adopted in a minority of patients. It is advisable that future and more comprehensive studies would provide clarification on the safety and efficacy of these regimens, especially in association with prior IVT.

Relevant information regarding this specific aspect of the endovascular treatment of acute TL will be likely provided by the ongoing Thrombectomy In TANdem Occlusion (TITAN) randomized clinical trial (https://clinicaltrials.gov/study/NCT03978988) [25].

In addition to the peri-procedural antiplatelet therapy, other baseline characteristics should be taken into consideration when assessing the risk of brain hemorrhage. Indeed, previous reports have highlighted the importance of a greater baseline ischemic core as predictor of intracranial hemorrhage after eCAS [7, 26]. Although this variable was among the covariates used in our matching algorithm, our cohort predominantly consisted of patients with a relatively small ischemic core, and it is possible that the results may differ in patients with lower ASPECTS.

Finally, our matched analysis showed no differences between the groups regarding rates of successful recanalization or long-term favorable clinical outcomes. This suggests a stronger effect of MT with eCAS on achieving recanalization, and is in line with previous studies showing no significant effect of IVT on functional outcomes in this patient population [2, 3, 16].

### Limitations

Our study has limitations, mainly deriving from its retrospective design. Although clinical and procedural records were carefully reviewed, the results could have been affected by the quality of data collected outside the strict criteria of a randomized trial. Uncontrolled biases may include variations in endovascular treatment protocols across centers and over the relatively long time of observation, which may have influenced the interpretation of results. Also, we cannot exclude the possibility that in some patients, the peri-procedural therapeutic choice was based on features relevant for the risk of bleeding or stent thrombosis that were not among the collected variables. The numerical imbalance between the different therapies may have influenced the results of their specific role on the occurrence of hemorrhagic transformation. Several other aspects that may also be predictive of short-term bleeding events were not available in our analysis, including the admission to an intensive care unit or a stroke unit, post-procedural blood pressure control, the extent of collateral circulation and the final volume of brain infarct. Additionally, other aspects of the post-exposure phase, such as the timing and dosage of post-procedural antithrombotic therapies, which may impact infarct progression and bleeding risk, were not part of our experimental design but are worth considering in future studies. Although the PSM method helps reducing biases in retrospective studies, the matching algorithm used in our analysis was centered on a set of covariates that we believe are relevant for the selected outcome measures, but it is possible that other factors may have been overlooked or missed, thus accounting for residual confounding. Also, this statistical approach resulted in a reduction of the size of individual samples, that may not be sufficient to detect meaningful differences and could not be applied across different antiplatelet regimens.

## Conclusions

Our multicenter matched analysis shows that the safety of MT with eCAS in patients with acute TL is not affected by prior administration of IVT. Therefore, intravenous thrombolysis should not be considered a factor withholding eCAS because of a perceived increased risk of intracerebral hemorrhage when combined with peri-procedural antiplatelet agents required for stent patency.

In addition, IVT is not associated with improved recanalization or clinical outcome. These results should be interpreted with caution and require confirmation by forthcoming randomized controlled trials to establish more definitive conclusions regarding the safety and efficacy of IVT in the context of TL acute endovascular management.

## Electronic supplementary material

Below is the link to the electronic supplementary material.

Supplementary Material 1

## Data Availability

No datasets were generated or analysed during the current study.
