# Supplementary material for: Safety of emergent carotid stenting after thrombolysis: a multicenter retrospective matched analysis
Source: Neuroradiology. 2025 Mar 21;67(4):979–85. doi: 10.1007/s00234-025-03571-8 (PMC12041103; doi:10.1007/s00234-025-03571-8)
Supplement: Supplementary file 1 — Supplementary Material 1 [file 234_2025_3571_MOESM1_ESM.docx]

**Safety of emergent carotid stenting after thrombolysis: a multicenter retrospective matched analysis**

**Supplementary Material**

| **Supplementary Table 1**  Demographic, baseline clinical and radiological parameters of the raw population of patients | | | | | |  |
| --- | --- | --- | --- | --- | --- | --- |
| **Variables** | **IVT**  **(N=265)** | | **no-IVT**  **(N=295)** | **p *** | | |
|  | |  |  |  | | |
|  | |  |  | |  | |
| ***Demographics*** | |  |  | |  | |
| sex (female), n/N (%) | | 72/265 (27.2%) | 102/295 (34.6%) | | 0.1 | |
| age, median (IQR) | | 67 (58-77) | 68 (58-76) | | 0.9 | |
|  | |  |  | |  | |
| ***Baseline clinical features and medical therapy*** | |  |  | |  | |
| hypertension, n/N (%) | | 150/265 (56.6%) | 178/295 (60.3%) | | 0.4 | |
| atrial Fibrillation, n/N (%) | | 15/265 (5.7%) | 30/295 (10.2%) | | **0.05** | |
| diabetes, n/N (%) | | 33/265 (12.0%) | 46/295 (15.6%) | | 0.3 | |
| dyslipidemia, n/N (%) | | 74/265 (27.9%) | 82/295 (27.8%) | | 0.9 | |
| coronary artery disease, n/N (%) | | 40/265 (15.1%) | 42/295 (14.2%) | | 0.8 | |
| glucose at baseline, mean ± SD, g/dl | | 130.6 ± 34.0 | 131.2 ± 40.3 | | 0.1 | |
| platelet count at baseline, mean ± SD, × 10^3^ | | 236.3 ± 73.2 | 226.6 ± 61.7 | | 0.8 | |
| INR at baseline, mean ± SD | | 1.05 ± 0.22 | 1.08 ± 0.35 | | 0.2 | |
| aPTT at baseline, mean ± SD, seconds | | 27.3 ± 6.4 | 27.3 ± 8.2 | | 0.2 | |
| previous stroke, n/N (%) | | 9/237 (3.9%) | 15/247 (6.1%) | | 0.2 | |
| anticoagulant at baseline, n/N (%) | | |  | | **< 0.001** | |
| none | | 260/265 (98.1%) | 268/295 (91.0%) | |  | |
| warfarin | | 5/265 (1.9%) | 23/295 (7.8%) | |  | |
| DOAC | | 0/265 (0.0%) | 4/295 (1.3%) | |  | |
| antiplatelet at baseline, n/N (%) | | 67/265 (25.3%) | 79/295 (26.8%) | | 0.7 | |
| statins, n/N (%) | | 59/254 (23.2%) | 67/288 (23.3%) | | 0.6 | |
| pre-stroke mRS score, median (IQR) | | 0 (0-0) | 0 (0-0) | | 0.3 | |
| baseline NIHSS score, median (IQR) | | 15 (10-19) | 15 (10-19) | | 0.9 | |
|  | |  |  | |  | |
| ***Baseline imaging data*** | |  |  | |  | |
| baseline ASPECTS, median (IQR) | | 9 (7-10) | 8 (7-10) | | **0.001** | |
| Fazekas score DWM, median (IQR) | | 1 (0-2) | 1 (0-2) | | 0.3 | |
| Fazekas score PVWM, median (IQR) | | 1 (0-1) | 1 (0-1) | | 0.3 | |
| cervical ICA pathology (atherothrombotic), n/N (%) | | 215/265 (81.1%) | 245/295 (83.0%) | | 0.6 | |
| site of occlusion | |  |  | |  | |
| ICA + M1-MCA | | 188/265 (71.0%) | 197/295 (66.8%) | | 0.8 | |
| ICA + M2-MCA | | 77/265 (29.0%) | 98/295 (33.2%) | | 0.2 | |
|  | |  |  | |  | |

IVT = intravenous thrombolysis; SD = Standard Deviation; INR = international normalized ratio; aPTT = partial thromboplastin time; DOAC = Direct oral anticoagulants; mRS = modified Rankin Scale; IQR = interquartile range; NIHSS = National Institute of Health Stroke Scale; ASPECTS = Alberta Stroke Program Early Computed Tomography score; DWM = deep white matter; PVWM = periventricular white matter; ICA = internal carotid artery; MCA = middle cerebral artery; * Statistical significance was considered at p < 0.05.

| **Supplementary Table 2**  Peri-procedural, post-procedural and outcome data of the raw population of patients | | | | | |
| --- | --- | --- | --- | --- | --- |
| **Variables** | **IVT** | **no-IVT** | **p *** | |  |
|  |  |  |  | |  |
|  |  |  | |  |  |
| ***Peri- and post-procedural data*** |  |  | |  |  |
|  |  |  | |  |  |
| onset-to-groin time, mean ± SD, minutes | 246.4 ± 115.6 | 280.8 ± 207.1 | | **< 0.001** |  |
| antiplatelet therapy for eCAS, n/N (%) | |  | | 0.1 |  |
| none | 4/265 (1.5%) | 8/295 (2.7%) | |  |  |
| i.v. aspirin (250-500 mg) | 62/265 (23.4%) | 57/295 (19.3%) | |  |  |
| i.v. aspirin (250-500 mg) and clopidogrel (75-500 mg) | 41/265 (15.5%) | 35/295 (11.9%) | |  |  |
| cangrelor (30 μg/kg bolus + 4 μg/kg/min i.v. for 2 hours) | 21/265 (7.9%) | 41/295 (13.9%) | |  |  |
| GPI loading dose (tirofiban 12 μg/kg in 30 min or  abciximab 0.25 mg/kg) | 9/265 (3.4%) | 11/295 (3.7%) | |  |  |
| cangrelor (30 μg/kg bolus + 4 μg/kg/min i.v. for 2 hours)  + GPI maintenance (tirofiban 0.1 μg/kg/min or abciximab  0.125 mg/kg/min) over 12 hours after eCAS | 6/265 (2.3%) | 3/295 (1.0%) | |  |  |
| i.v. aspirin (250-500 mg) + GPI maintenance (tirofiban 0.1  μg/kg/min or abciximab 0.125 mg/kg/min) over 12 hours  after eCAS | 20/265 (7.5%) | 31/295 (10.5%) | |  |  |
| GPI loading dose (tirofiban 12 μg/kg in 30 min or  abciximab 0.25 mg/kg) + GPI maintenance (tirofiban 0.1  μg/kg/min or abciximab 0.125 mg/kg/min) over 12 hours  after eCAS | 101/265 (38.1%) | 103/295 (34.9%) | |  |  |
| i.v. aspirin (250-500 mg) and clopidogrel (75-500 mg) +  GPI maintenance (tirofiban 0.1 μg/kg/min or abciximab 0.125  mg/kg/min) over 12 hours after eCAS | 1/265 (0.4%) | 6/295 (2.0%) | |  |  |
|  | |  | |  |  |
|  |  |  | |  |  |
| mTICI score 2b-3, n/N (%) | 224/261 (85.8%) | 257/291 (88.3%) | | 0.4 |  |
|  |  |  | |  |  |
| ***Procedure-related and post-procedural adverse events*** |  |  | |  |  |
| intraprocedural embolism, n/N (%) | 26/234 (11.1%) | 42/274 (15.3%) | | 0.2 |  |
| intraprocedural dissection, n/N (%) | 9/234 (3.8%) | 13/272 (4.8%) | | 0.7 |  |
| early stent thrombosis, n/N (%) | 30/265 (11.3%) | 31/295 (10.5%) | | 0.81 |  |
|  |  |  | |  |  |
| ***Safety and clinical outcome data*** |  |  | |  |  |
| PH type 2, n/N (%) | 19/265 (7.2%) | 24/295 (8.1%) | | 0.7  7 |  |
| PH type 1, n/N (%) | 65/265 (24.5%) | 73/295 (24.7%) | | 0.9 |  |
| major extracranial bleeding, n/N (%) | 3/234 (1.3%) | 1/274 (0.3%) | | 0.2 |  |
| 90-day mRS 0-2, n/N (%) | 157/247 (63.6%) | 141/258 (54.6%) | | **0.04** |  |
| 90-day mortality, n/N (%) | 28/247 (11.3%) | 33/258 (12.8%) | | 0.6 |  |
|  |  |  | |  |  |

IVT = intravenous thrombolysis; SD = standard deviation, eCAS = emergent carotid artery stenting; GPI = Glycoprotein IIb/IIIa inhibitor; mTICI = modified Treatment In Cerebral Infarction; PH = parenchymal hemorrhage; mRS = modified Rankin Scale; * Statistical significance was considered at p < 0.05.

**Supplementary Table 3**

Standardized mean difference of covariates before and after PSM of the entire population of patients

|  |  | | |  | |  |  |  |  | |
| --- | --- | --- | --- | --- | --- | --- | --- | --- | --- | --- |
|  | | **Pre-match population** | | | | | **Post-match population** | | | |
| **Variables** | | **IVT**  **(N=265)** | | | **no-IVT**  **(N=295)** | **SMD *** | **IVT**  **(N=174)** | **no-IVT**  **(N=174)** | | **SMD *** |
|  | |  | | |  |  |  |  | |  |
| age, mean ± SD, years | | 66.9 ± 12.9 | | | 66.5 ± 13.0 | **0.03** | 67.3 ± 12.6 | 65.5 ± 13.1 | | **0.05** |
| NIHSS score, median (IQR) | | 15 (10-19) | | | 15 (10-19) | **0.02** | 15 (9-18) | 15 (9-18) | | **0.03** |
| baseline ASPECTS, median (IQR) | | 8 (7-10) | | | 7 (7-10) | 0.3 | 9 (7-10) | 9 (7-10) | | **0.08** |
| antiplatelet at baseline, n/N (%) | | 67/265 (25.3%) | | | 79/295 (26.8%) | **-0.02** | 42/174 (24.1%) | 41/174 (23.6%%) | | **0.01** |
| anticoagulant at baseline, n/N (%) | |  | | |  | -0.3 |  |  | | **0.09** |
| none | | 260/265 (98.1%) | | | 268/295 91.0%) |  | 169/174 (97.1%) | 173/174 (99.4%) | |  |
| warfarin | | 5/265 (1.9%) | | | 23/295 (7.8%) |  | 5/174 (2.9%) | 1/174 (0.5%) | |  |
| DOAC | | 0/265 (0.0%) | | | 4/295 (1.3%) |  | 0/174 (0.0%) | 0/174 (0.0%) | |  |
| antiplatelet therapy for eCAS, n/N (%) | | |  | |  | **0.04** |  |  | | **-0.01** |
| i.v. aspirin | | 62/265 (23.4%) | | | 57/295 (19.3%) |  | 38/174 (21.8%) | 40/174 (23.0%) | |  |
| i.v. aspirin + clopidogrel | | 41/265 (15.5%) | | | 35/295 (11.9%) |  | 23/174 (13.2%) | 23/174 (13.2%) | |  |
| cangrelor | | 21/265 (7.9%) | | | 41/295 (13.9%) |  | 18/174 (10.3%) | 16/174 (9.2%) | |  |
| GPI loading dose | | 9/265 (3.4%) | | | 11/295 (3.7%) |  | 7/174 (4.0%) | 5/174 (2.9%) | |  |
| cangrelor + GPI maintenance | | 6/265 (2.3%) | | | 3/295 (1.0%) |  | 3/174 (1.7%) | 2/174 (1.1%) | |  |
| i.v. aspirin + GPI maintenance | | 20/265 (7.5%) | | | 31/295 (10.5%) |  | 17/174 (9.8%) | 14/174 (8.0%) | |  |
| GPI loading dose + GPI maintenance | | 101/265 (38.1%) | | | 103/295 (34.9%) |  | 65/174 (37.4%) | 67/174 (38.5%) | |  |
| i.v. aspirin + clopidogrel + GPI  maintenance | | 1/265 (0.4%) | | | 6/295 (2.0%) |  | 1/174 (0.6%) | 2/174 (1.1%) | |  |
| none | | 4/265 (1.5%) | | | 8/295 (2.7%) |  | 2/174 (1.1%) | 5/174 (2.9%) | |  |
| onset-to-groin time, mean ± SD, minutes | | 246.43 ± 115.6 | | | 280.9 ± 207.1 | -0.2 | 249.2 ± 121.8 | 261.9 ± 166.9 | | **-0.09** |
|  | |  | | |  |  |  |  | |  |
|  | |  | | |  |  |  |  | |  |

PSM = Propensity Score Matching; IVT = intravenous thrombolysis; SMD = standardized mean difference; SD = Standard Deviation; NIHSS = National Institutes of Health Stroke Scale; IQR = interquartile range; ASPECTS = Alberta Stroke Program Early Computed Tomography score; DOAC = direct oral anticoagulants; eCAS = emergent carotid artery stenting; GPI = glycoprotein IIb/IIIa inhibitor; * SMD was calculated for each covariate to verify the balance between treatment groups, SMD < 0.1 indicates negligible imbalance between treatment groups.

**Supplementary Table 4**

Distribution of the different intra-procedural antiplatelet therapies in the post-match population

| **Antiplatelet regimen** | | **IVT**  **(N = 174)** | | **no-IVT**  **(N=174)** | |
| --- | --- | --- | --- | --- | --- |
|  |  | |  | |  |
| no antiplatelet therapy, n/N (%) | |  | 2/174 (1.1%) | | 5/174 (2.9%) |
| i.v. aspirin, n/N (%) | |  | 38/174 (21.8%) | | 40/174 (23%) |
| i.v. aspirin + clopidogrel, n/N (%) | |  | 23/174 (13.2%) | | 23/174 (13.2%) |
| cangrelor, n/N (%) | |  | 18/174 (10.3%) | | 16/174 (10.3%) |
| GPI loading dose, n/N (%) | |  | 7/174 (4.0%) | | 5/174 (2.9%) |
| cangrelor + GPI maintenance, n/N (%) | |  | 3/174 (1.7%) | | 2/174 (1.1%) |
| i.v. aspirin + GPI maintenance, n/N (%) | |  | 17/174 (9.8%) | | 14/174 (8.0%) |
| GPI loading dose + GPI maintenance, n/N (%) | |  | 65/174 (37.3%) | | 67/174 (38.5%) |
| i.v. aspirin + clopidogrel + GPI maintenance, n/N (%) | |  | 1/174 (0.5%) | | 2/174 (1.1%) |
|  | |  |  | |  |

IVT = intravenous thrombolysis; GPI = glycoprotein IIb/IIIa inhibitor.

**Supplementary Table 5**

Standardized mean difference of covariates at baseline before and after PSM in patients receiving peri-procedural i.v. aspirin

|  |  | | |  | |  |  | |  |  | |
| --- | --- | --- | --- | --- | --- | --- | --- | --- | --- | --- | --- |
|  | | **Pre-match population** | | | | | **Post-match population** | | | | |
| **Variables** | | **IVT**  **(N=62)** | **no-IVT**  **(N=57)** | | | **SMD *** | **IVT**  **(n=26)** | **no-IVT**  **(n=26)** | | | **SMD *** |
|  | |  | | |  |  |  | |  | |  |
| age, mean ± SD, years | | 67.8 ± 12.2 | | | 66.0 ± 12.2 | 0.15 | 70.9 ± 10.6 | | 64.1 ± 12.8 | | 0.56 |
| NIHSS score, median (IQR) | | 16 (12-21) | | | 15 (11-21) | 0.21 | 15 (10-19) | | 17 (10-20) | | -0.31 |
| baseline ASPECTS, median (IQR) | | 8 (7-9) | | | 8 (7-9) | 0.10 | 8 (7-10) | | 8 (7-10) | | **-0.09** |
| antiplatelet at baseline, n/N (%) | | 13/62 (21.0%) | | | 17/57 (29.8%) | **-0.09** | 8/26 (30.8%) | | 9/26 (34.6%) | | **-0.04** |
| anticoagulant at baseline, n/N (%) | |  | | |  | **-0.09** |  | |  | | **0.00** |
| none | | 62/62 (100.0%) | | | 52/57 (91.2%) |  | 26/26 (100.0%) | | 26/26 (100.0%) | |  |
| warfarin | | 0/62 (0.0%) | | | 5/57 (9.0%) |  | 0/26 (0.0%) | | 0/26 (0.0%) | |  |
| DOAC | | 0/62 (0.0%) | | | 0/57 (0.0%) |  | 0/26 (0.0%) | | 0/26 (0.0%) | |  |
| onset-to-groin time, mean ± SD, minutes | | 266.5 ± 123.9 | | | 353.3 ± 256.9 | -0.43 | 270.8 ± 121.1 | | 260.0 ± 153.5 | | **0.09** |
|  | |  | | |  |  |  | |  | |  |
|  | |  | | |  |  |  | |  | |  |

PSM = Propensity Score Matching; IVT = intravenous thrombolysis; SMD = standardized mean difference; SD = Standard Deviation; NIHSS = National Institutes of Health Stroke Scale; IQR = interquartile range; ASPECTS = Alberta Stroke Program Early Computed Tomography score; DOAC = direct oral anticoagulants; * SMD was calculated for each covariate to verify the balance between treatment groups, SMD < 0.1 indicates negligible imbalance between treatment groups.

**Supplementary Table 6**

Standardized mean difference of covariates at baseline before and after PSM in patients receiving peri-procedural i.v. GPI loading dose plus GPI maintenance

|  |  | |  | |  |  | |  |  | |
| --- | --- | --- | --- | --- | --- | --- | --- | --- | --- | --- |
|  | | **Pre-match population** | | | | **Post-match population** | | | | |
| **Variables** | | **IVT**  **(N=101)** | **no-IVT**  **(N=103)** | | **SMD *** | **IVT**  **(N=54)** | **no-IVT**  **(N=54)** | | | **SMD *** |
|  | |  | |  |  |  | |  | |  |
| age, mean ± SD, years | | 65.6 ± 14.1 | | 64.7 ± 14.7 | **0.01** | 65.6 ± 14.3 | | 62.6 ± 15.0 | | 0.21 |
| NIHSS score, median (IQR) | | 15 (10-19) | | 16 (11-20) | **-0.09** | 16 (10-19) | | 16 (10-18) | | **0.09** |
| baseline ASPECTS, median (IQR) | | 8 (7-10) | | 7 (6-9) | 0.50 | 8 (7-9) | | 7 (7-10) | | **-0.03** |
| antiplatelet at baseline, n/N (%) | | 23/101 (22.8%) | | 24/103 (23.3%) | **-0.01** | 11/54 (20.4%) | | 10/54 (18.5%) | | **0.02** |
| anticoagulant at baseline, n/N (%) | |  | |  | -0.24 |  | |  | | **-0.09** |
| none | | 97/101 (96.0%) | | 94/103 (91.2%) |  | 52/54 (96.3%) | | 51/54 (94.4%) | |  |
| warfarin | | 4/101 (4.0%) | | 6/103 (5.8%) |  | 2/54 (3.7%) | | 1/54 (1.8%) | |  |
| DOAC | | 0/101 (0.0%) | | 3/103 (2.9%) |  | 0/54 (0.0%) | | 1/54 (1.8%) | |  |
| onset-to-groin time, mean ± SD | | 253.0 ± 124.5 | | 279.4 ± 177.3 | -0.17 | 252.8 ± 132.7 | | 255.1 ± 115.4 | | **-0.02** |
|  | |  | |  |  |  | |  | |  |
|  | |  | |  |  |  | |  | |  |

PSM = Propensity Score Matching; GPI = glycoprotein IIb/IIIa inhibitor; IVT = intravenous thrombolysis; SMD = standardized mean difference; SD = Standard Deviation; NIHSS = National Institutes of Health Stroke Scale; IQR = interquartile range; ASPECTS = Alberta Stroke Program Early Computed Tomography score; DOAC = direct oral anticoagulants; * SMD was calculated for each covariate to verify the balance between treatment groups, SMD < 0.1 indicates negligible imbalance between treatment groups.

**Supplementary Table 7**

Univariate analysis of patients under less frequent regimens of peri-procedural antiplatelet therapy

**i.v. aspirin + clopidogrel**

|  | | | | | |
| --- | --- | --- | --- | --- | --- |
|  | **IVT**  **(N=41)** | **no-IVT**  **(N=35)** | |  | **p value *** |
|  |  | |  |  |  |
| PH type 2, n/N (%) | 3/41 (7.3%) | | 2/35 (5.7%) |  | 1.0 |
| 90-day mortality, n/N (%) | 4/41 (9.7%) | | 2/34 (5.9%) |  | 0.7 |
|  |  | |  |  |  |
| PH type 1, n/N (%) | 6/41 (14.6%) | | 9/35 (25.7%) |  | 0.3 |
| major extracranial bleeding events, n/N (%) | 0/41 (0.0%) | | 0/35 (0.0%) |  | - |
| stent thrombosis <24h, n/N (%) | 2/41 (4.8%) | | 2/35 (5.7%) |  | 1.0 |
|  |  | |  |  |  |
| mTICI score 2b-3, n/N (%) | 39/41 (95.1%) | | 34/35 (97.1%) |  | 1.0 |
| 90-day mRS score 0-2, n/N (%) | 34/41 (82.9%) | | 18/34 (52.9%) |  | **0.01** |
|  |  | |  |  |  |
|  |  | |  |  |  |

**i.v. aspirin + GPI maintenance**

|  | | | | | |
| --- | --- | --- | --- | --- | --- |
|  | **IVT**  **(N=20)** | **no-IVT**  **(N=31)** | |  | **p value *** |
|  |  | |  |  |  |
| PH type 2, n/N (%) | 1/20 (5.0%) | | 7/31 (22.6%) |  | 0.1 |
| 90-day mortality, n/N (%) | 5/20 (25.0%) | | 7/31 (22.6%) |  | 1.0 |
|  |  | |  |  |  |
| PH type 1 n/N (%) | 12/20 (60.0%) | | 12/31 (38.7%) |  | 0.2 |
| major extracranial bleeding events, n/N (%) | 0/20 (0.0%) | | 0/31 (0.0%) |  | - |
| stent thrombosis <24h, n/N (%) | 2/20 (10.0%) | | 4/31 (12.9%) |  | 1.0 |
|  |  | |  |  |  |
| mTICI score 2b-3, n/N (%) | 12/20 (60.0%) | | 27/31 (87.1%) |  | **0.04** |
| 90-day mRS score 0-2, n/N (%) | 10/20 (50.0%) | | 14/31 (45.1%) |  | 0.8 |
|  |  | |  |  |  |
|  |  | |  |  |  |

**i.v. cangrelor**

|  | | | | | |
| --- | --- | --- | --- | --- | --- |
|  | **IVT**  **(N=21)** | **no-IVT**  **(N=41)** | |  | **p value *** |
|  |  | |  |  |  |
| PH type 2, n/N (%) | 0/21 (0.0%) | | 1/41 (2.4%) |  | 1.0 |
| 90-day mortality, n/N (%) | 0/7 (0.0%) | | 2/9 (22.2%) |  | 0.5 |
|  |  | |  |  |  |
| PH type 1, n/N (%) | 4/21 (19.0%) | | 3/41 (7.3%) |  | 0.2 |
| major extracranial bleeding events, n/N (%) | 0/21 (0.0%) | | 0/41 (0.0%) |  | - |
| stent thrombosis < 24h, n/N (%) | 0/21 (0.0%) | | 1/41 (2.4%) |  | 1.0 |
|  |  | |  |  |  |
| mTICI score 2b-3, n/N (%) | 18/19 (94.7%) | | 35/38 (92.1%) |  | 1.0 |
| 90-day mRS score 0-2, n/N (%) | 6/7 (85.7%) | | 7/9 (77.8%) |  | 1.0 |
|  |  | |  |  |  |
|  |  | |  |  |  |

IVT = intravenous thrombolysis; PH = parenchymal hemorrhage; mTICI = modified Treatment In Cerebral Infarction; mRS = modified Rankin Scale; GPI = glycoprotein IIb/IIIa inhibitor; * statistical significance was considered at p < 0.05.
